# 

**Published:** 1923-04

**Authors:** 


**THE DENTAL REGISTER** is published every month by the Samuel A. Crocker Company, 18 W. 7th Street, Cincinnati, O. It is mailed to subscribers during the last week in each month, being a month-end publication. Have copy for current month here by the 15th of each month. Copy received by the 15th of the month goes into that month's number of the Dental Register. Publication and business office, 18 W. 7th Street, Cincinnati, Ohio. Editorial office, 18 W. 7th Street, Cincinnati, Ohio. Address all correspondence to Editor Dental Register, Box 838, Cincinnati, Ohio, c'o The Samuel A. Crocker Company.

Notices of meetings, announcements of interest to the dental profession, and particulars of new ideas and new inventions will be given free publication in reading section. Advertising section is open to all ethical advertisements.

We are not responsible for opinions or statements made by writers whose articles this magazine may publish—nor does the publishing of any article infer in any way that we indorse the views or opinions expressed by any writer whose article we may publish.

---

Sal Hepatica

Materially Aids
Local Treatment

IN

PYORRHEA

Samples on Request

Bristol - Myers Co.

NEW YORK

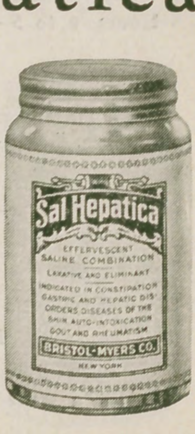

### DIRECTORY

FOR

### Members of the Dental Profession whose practices are limited to a specialty.

The object of this directory is to furnish ready reference to specialists who take referred patients. If you wish your name inserted in this list please correspond with the Dental Register, Box 838, Cincinnati, Ohio.

Your name, specialty, office hours and phone number will be published.

This entire page is reserved for Professional Directory.

### Cincinnati

Dental X-Ray Diagnosis
CHARLES K. FIELD
61 Groton Bldg., 7th and Race Sts.
Hours, 10 to 4
Canal 2127

Periodontoclasia (Pyorrhea and Oral Prophylaxis)
EDGAR H. EBERLE
605-06 Union Central Bldg., 4th and Vine Sts.
Hours, 9 to 5—Consultation hours by appointment
Main 882

Periodontoclasia (Pyorrhea and Oral Prophylaxis)
MARIE L. BECKERT
25 Groton Bldg., 7th and Race Sts.
Hours, 9 to 5—Consultation hours by appointment
Canal 842

Exodontia (Extraction and Oral Surgery)
ROBERT M. SCHELL
20 W. 9th St., between Vine and Race Sts.
Hours, 1 to 5—Consultation by appointment
Canal 118

### ANNOUNCEMENTS

### MASSACHUSETTS DENTAL SOCIETY

The fifty-ninth Annual Meeting of the Massachusetts Dental Society will be held at the Copley-Plaza Hotel, Boston, Mass., May 2-4, 1923.

The Executive Committee of the Society wish to extend a cordial invitation to all ethical dentists to attend the meeting.

The Councilors' meeting for transaction of Society business will be held on Tuesday, May 1st. (Hour to be announced later.)

Wednesday evening will be devoted to a reception, entertainment and dance.

The large Ballroom of the Hotel will be brimming full of Exhibits.

The meeting will be conducted under four sections. Lectures and Teaching clinics will be provided by each section. Lectures will be open. Admission to the Teaching clinics will be by special tickets only.

*Teaching clinics in Operative Section:*

By T. W. Maves, D. D. S., University of Minnesota, Minn.; William Dwight Tracy, D. D. S., New York City, and others.

*Teaching clinics in Prosthetic Section:*

By M. M. House, D. D. S., Deaner Dental Institute, Kansas City, Mo.; W. E. Cummer, D. D. S., Royal College of Dental Surgeons, Toronto, Ont.

*Teaching clinics in Oral Surgery Section:*

By Arthur E. Smith, D. D. S., M. D., Chicago, Ill.; Clarence O. Simpson, M. D., D. D. S., St. Louis, Mo.

*Teaching clinics in Preventive Dentistry Section:*

By John Oppie McCall, A. B., D. D. S., Columbia University, N. Y.; Wallace Seccombe, F. A. C. D., Royal College of Dental Surgeons, Toronto, Ont., and others.

Members who wish to apply for tickets to the Teaching clinics will be charged a registration fee of $2.00 for each section, which will entitle them to all Teaching clinics under the section.

Nonmembers who wish to apply for tickets will be charged a registration fee of $10.00 in addition to the regular section registration fees.

For application to Teaching clinics apply to the Secretary.

Reduced railroad rates will be granted by the New England Passenger Association from points in New England States and from Albany and New York City in New York.—W. Vernon Ryder, Secretary, 175 Newbury St., Boston, Mass.

---

### WEST VIRGINIA STATE DENTAL SOCIETY

The seventeenth Annual Meeting of the West Virginia State Dental Society will be held May 21-23, 1923, at Wheeling, W. Va. Headquarters, Hotel McLure

### OFFICERS AND COMMITTEES FOR 1923

*Officers*—G. C. Howard, President, West Union; F. E. Hess, First Vice-President, Fairmont; S. E. Langfitt, Second Vice-President, Huntington; N. P. MacDermid, Secretary, Charleston; J. S. Stone, Treasurer, Clarksburg.

*National Delegates*—H. W. Burnett, Fairmont; H. T. Halstead, Huntington; S. T. Bird, Princeton.

*Alternate Delegates*—E. Earl McCray, Fairmont; S. M. Callaway, Huntington; J. C. Houchins, North Fork.

*Program*—Roy C. Loudin, Chairman, Moundsville; Frank L. Wright, Wheeling; A. E. Hennon, Wheeling; F. M. Farnsworth, Buckhannon.

*Organization*—C. H. Layman, Chairman, Fairmont; A. I. Marple, Huntington; W. E. Paul, Richwood; J. D. Morford, Sistersville; N. McK. Wilson, Davis.

*Oral Hygiene and Publicity*—G. T. Epling, Chairman, Welch; Roy C. Loudin, Moundsville; C. H. Neill, Fairmont; H. H. Ramsey, Mt. Hope; P. F. Schaffer, Charleston.

*Necrology*—L. A. Stark, Chairman, Shinnston; C. R. Singleton, Charleston; H. H. Stoops, Pennsboro; S. T. Bird, Princeton; C. S. Coffman, Lewisburg.

*Ethics*—L. G. Beerbower, Chairman, Terra Alta; C. H. Steinbeck, Parkersburg; H. C. Hill, Logan; H. A. Duncan, Oak Hill; H. E. Summers, Huntington.

*Legislation*—H. H. Smallridge, Chairman, Charleston; H. L. Satterfield, Fairmont; L. L. Belcher, Welch; L. J. Walker, Clarksburg.

*Executive Council*—G. C. Howard, Chairman, West Union; W. S. Rosenheim, Williamson; J. N. Mahan, Charleston; F. E. Hess, Fairmont; S. E. Langfitt, Huntington; N. P. MacDermid, Charleston; J. S. Stone, Clarksburg; H. J. Scott, Clarksburg; H. W. Black, Bluefield; F. N. Carroll, Wheeling; A. G. Eisenman, Huntington; W. T. Riffe, Beckley; C. M. Jividen, Charleston; C. S. Lytle, Parkersburg; H. C. Hill, Logan; C. S. Coffman, Lewisburg; G. B. Geyer, Martinsburg.

*Arrangements*—(Wheeling Members of the Wheeling District Dental Society.) J. H. McClure, Chairman, Wheeling; O. W. Burdats, Wheeling; E. S. Bullard, Wheeling; W. E. H. Caldwell, Wheeling; Alvin B. Cummings, Wheeling; F. N. Carroll, Wheeling; A. E. Hennon, Wheeling; W. L. Hogue, Wheeling; W. R. Loper, Wheeling; John L. Dunn, Wheeling; W. B. McKee, Wheeling; Wm. F. McKinley, Wheeling; C. Bates McLain, Wheeling; A. C. Plant, Wheeling; O. L. Richmond, Wheeling; J. A. Vanyo, Wheeling; Frank L. Wright, Wheeling; B. Lawrence Riggs, Wheeling; H. W. Gatewood, Wheeling; John G.

Arbenz, Wheeling; John W. Storer, Wheeling; J. J. Carroll, Wheeling.

*Membership*—H. J. Scott, Chairman, Secretary "Monongahela Valley Dental Society," Clarksburg; A. G. Eisenman, Secretary "Huntington Dental Society," Huntington; H. W. Black, Secretary "Mercer-Mingo-McDowell Dental Society," Bluefield; F. N. Carroll, Secretary "Wheeling District Dental Society," Wheeling; W. T. Riffe, Secretary "New River Dental Society," Beckley; C. S. Lytle, Secretary "Parkersburg Dental Society," Parkersburg; C. M. Jividen, Secretary "Kanawha Valley Dental Society," Charleston; H. C. Hill, Secretary "Logan County Dental Society," Logan; C. S. Coffman, Secretary "Greenbrier Valley Dental Society," Lewisburg; G. B. Geyer, Secretary "Potomac Dental Society," Martinsburg.

A large attendance is expected and it is hoped to make this the best meeting ever held in the State. There is an excellent room for the exhibitors, which will be held both day and night, with every convenience. 110 V. A.C. for those who will need electricity.

The sessions of the society will be held in a separate room, so there will not be any conflict between meetings and exhibitors.

One evening will be given entirely to the exhibitors, no meeting at that time.

---

### DENTIST NINETY-NINE YEARS OLD DIES

Cleveland, which will soon become the center of dental activities because of the American Dental Association Meeting in September, has had to stop and reflect upon the life of one of its pioneer dentists, who died at the age of ninety-nine years, having practiced seventy years.

Dr. William Perry Horton, long known in dentistry as an energetic practitioner and a citizen active in furthering the best interests of the community, was born in

Pittsford, Vt., October 28, 1823. He came to Cleveland in 1844, when this city was but eight years old, with about 10,000 inhabitants. He later attended Oberlin College, returning to Cleveland in 1851 and forming a partnership with Dr. Benjamin Strickland, who had established himself as the city's first dentist in 1835.

Doctor Horton's active practice covered a period of nearly seventy years, which, perhaps, constitutes a record.

When the Northern Ohio Dental Association was organized in 1857, Doctor Horton became a charter member of it. He took an active part in the formation of the Ohio State Dental Society, becoming the first president of that organization.

In 1869 he was elected to the Cleveland city council from the old sixth ward and served for a period of eight years. He was also a member of the historic centennial council of thirty-six members.

Doctor Horton, formerly a Whig in politics, was one of the seven men who organized the Republican party. Years later he became a Progressive.

The widow, Mrs. Margaret Horton; three grandchildren and four great-grandchildren survive.

---

### A PIONEER DENTIST

The American Dental Association, which meets in Cleveland next September, will honor the memory of one who was a real pioneer in dentistry.

Dr. Charles Richard Butler was a Cleveland product, and among the many Presidents of the American Dental Association, became one of the most noted dentists of his time.

His inventive genius and capabilities were shining examples of the qualities of those who pioneered their way into the fields that made dentistry a real profession.

Doctor Butler was born in 1832 and came to Cleveland in 1854, where he studied dentistry with Dr. William H.

Atkinson and Dr. M. L. Wright. He also studied surgery with a noted surgeon, Dr. Elisha Sterling.

In 1858 he graduated from the Pennsylvania College of Dental Surgery and in 1865 from the Cleveland Medical College.

He was the first Professor of Clinical Operative Dentistry in the country, the ''chair'' being created for him by the Ohio College of Dental Surgery in 1867. Here also he taught Steel Technics, as he was an adept at handling steel. When he went to dental college in 1857 he took pluggers and excavators of his own manufacture and a mallet for condensing cohesive gold foil. He was called the ''hammer and punch man.'' In 1860 he made a mallet of a cylinder of laurel-root filled with tin and lead melted together. The handle was of live oak taken from the battleship Kearsarge when it was dismantled. Before the dental engine was used (1868) he made a set of burs for cutting down fillings.

In 1868 he made a set of fifteen hand mallet pluggers; in 1878 another set of eight. He invented and made many excavators, chisels and other instruments.

He was one of the finest operators and being satisfied only with the best, caused him to manufacture what was needed if it could not be purchased. This was true in many ways, both in and out of dentistry. He became an expert in precious stones which were mounted with great skill in beautiful settings that he designed.

He was probably the first in Cleveland to give much of any attention to the ''correction of irregularities of the teeth'' in which he was successful.

Doctor Butler received many honors in his life and always was an honor to the trust that he held.

At Louisville, Ky., in August, 1888, he was elected President of the American Dental Association; was also President of the Ohio State Society, Northern Ohio Dental Association and Cleveland Dental Society. At the end of fifty-four years practice he was given a complimentary dinner by many dentists from Cleveland and other places.

When the dental department of the Western Reserve University was organized in 1892, Doctor Butler was one of the organizers and its first Dean.

He was an indefatigable worker in the Methodist church, held many offices of trust in this and the Masonic bodies, in which he was a distinguished 33° Mason. Doctor Butler was a friend of the young man in dentistry and was ever ready to lend them a helping hand.

Doctor Butler was born in Portage County, Ohio, in 1832 and died December 14, 1914, aged eighty-two years. His funeral was conducted by the Knights Templar amid a large encourse of friends.—W. H. Whitslar.

NOTE—The writer is indebted to H. L. Amblers' History of Dentistry in Cleveland for some of the above data.

---

### LIST OF EXHIBITORS AT THE KENTUCKY STATE DENTAL MEETING, APRIL 16, 1923

The American Cabinet Co., Two Rivers, Wis; Bristol-Myers Co., Brooklyn, N. Y.; Bates Hendershot Co., Louisville, Ky.; The Samuel A. Crocker Co., Cincinnati, Ohio; Colgate & Company, New York, N. Y.; L. D. Caulk Co., Milford, Del.; The Clarke Chemical Co., Wickliffe, Ohio; Wilmoth Castle Co., Rochester, N. Y.; T. M. Crutcher Dental Depot, Louisville, Ky.; Denver Chemical Co., New York City; The Dentist's Supply Co., New York; Edward X-ray Corp., Indianapolis, Ind.; Harmeyer & Brand Co., Cincinnati, Ohio; Horlick's Malted Milk Co., Racine, Wis.; The Heidbrink Co., Minneapolis, Minn.; The Harvard Co., Canton, Ohio; Kress & Owen Co., New York; Kolynos Company, New Haven, Conn.; Lavoris Chemical Co., Minneapolis, Minn.; Lambert Pharmacal Co., St. Louis, Mo.; H. R. Lathrop Co., New York, N. Y.; The Medical Protective Co., Fort Wayne, Ind.; Pepsodent Co., Chicago, Ill.; C. H. Phillips Chemical Co., New York, N. Y.; Poloris

Company, New York City; Ritter Dental Mfg. Co., Rochester, N. Y.; The Rich-Alloy Co., Cleveland, Ohio; Rinehart-Childs Co., Louisville, Ky.; Lee S. Smith & Son Mfg. Co., Pittsburgh, Pa.; Stratford-Cookson Co., Philadelphia, Pa.; The Sodiphene Co., Kansas City, Mo.; E. R. Squibb & Sons, New York; The Senreco Co., Cincinnati, Ohio; Toledo Dental Supply Co., Toledo, Ohio; Victor X-ray Corp., Chicago, Ill.; Walling Bros., Springville, N. Y.; The Webber Dental Mfg. Co., Canton, Ohio; H. B. Wiggin's Sons Co., Bloomfield, N. J.; Wagners Laboratory, Louisville, Ky.; The Louisville Dental Depot, Louisville, Ky.; S. S. White Dental Mfg. Co., Philadelphia, Pa.

---

"A WORD FROM THE SENATOR"

Chicago, Illinois.

*To the Graduate:*

What's going to bring your second patient to you? Your work or equipment? Those two things are the important factors in practice building.

You, no doubt, will begin work on your first patient just as soon as you hang out your shingle. You, probably, have that patient already booked. But, it is going to be some time before your good work on that patient proves itself.

So, that patient is not going to say much about your work right off the reel, but will surely say something to somebody about your equipment as soon as she gets out of your office; that's human nature.

With our twenty-five years experience in the dental industry, building equipment, we have always reckoned with the psychology of the dentist's clientele and know that the ornamental must be built into dentists' fixtures along with the mechanical and practical.

Of course, no dentist buys a thing for its beauty alone, but everyone likes to win the admiration of those he serves and we are positive that if you instal a Clark Unit Pedestal it will not be long before you realize that it is the most talked of fixture in your office.

On the other hand, for unified equipment of extreme moderate price, the Clark Clinic Pedestal in its simple, modest form provides exceptional value for the money and compels extraordinary admiration for a fixture of that class.

A mere combination of Engine, Spittoon, Table, etc., did not, we felt, constitute an ideal Unit any more than a diploma makes a good Dentist. We strove for a practical co-ordination of only vital Utilities to effect their operation with mechanical and electrical precision and to fashion them harmoniously.

The Clark Unit Pedestal of today, with its removable panels, reveals an unobstructed, roomy interior, easy of installation, free of electrical complications, simple to maintain, is built in a way that has elicited the praise of electrical and mechanical engineers, while the Clark Clinic Pedestal in its less comprehensive form reflects simplicity in its every detail with appearance that adds refinement and dignity to your operating room.

Put in a Clark Unit Pedestal or a Clark Clinic Pedestal, to facilitate your work and start the way to your own personal pride of ownership and the admiration of your clientele.—Yours respectfully, A. C. Clark & Co.

